# Transcreation: an implementation science framework for community-engaged behavioral interventions to reduce health disparities

**DOI:** 10.1186/s12913-018-3521-z

**Published:** 2018-09-12

**Authors:** Anna María Nápoles, Anita L. Stewart

**Affiliations:** 1 National Institute on Minority Health and Health Disparities, 9000 Rockville Pike, Building 3, Floor 5, Room E08, Bethesda, MD 20892 USA; 2 University of California San Francisco, 3333 California Street, Suite 350E, San Francisco, CA 94118 USA

**Keywords:** Evidence-based interventions, Intervention adaptation, Translation, Health disparities, Community-engaged research, Implementation science

## Abstract

**Background:**

Methods for translating evidence-based behavioral interventions into real-world settings seldom account for the special issues in reaching health disparity populations.

**Main text:**

The objective of this article is to describe an innovative “transcreational” framework for designing and delivering interventions in communities to reduce health disparities. We define transcreation as the process of planning, delivering, and evaluating interventions so that they resonate with the community experiencing health disparities, while achieving intended health outcomes. The Transcreation Framework for Community-engaged Behavioral Interventions to Reduce Health Disparities comprises seven steps: 1) identify community infrastructure and engage partners; 2) specify theory; 3) identify multiple inputs for new program; 4) design intervention prototype; 5) design study, methods, and measures for community setting; 6) build community capacity for delivery; and 7) deliver transcreated intervention and evaluate implementation processes. Communities are engaged from the start and interventions are delivered by community-based interventionists and tested in community settings. The framework applies rigorous scientific methods for evaluating program effectiveness and implementation processes. It incorporates training and ongoing technical assistance to assure treatment fidelity and build community capacity.

**Conclusions:**

This framework expands the types of scientific evidence used and balances fidelity to evidence and fit to the community setting. It can guide researchers and communities in developing and testing behavioral interventions to reduce health disparities that are likely to be sustained because infrastructure development is embedded in the research.

## Background

Health disparities in the United States (U.S.) based on race, ethnicity, socioeconomic status, geography, gender, sexual orientation, and disability status continue to persist. According to the Centers for Disease Control and Prevention (CDC) Health Disparities and Inequalities Report—U.S. 2013, the risk of premature death due to cardiovascular disease, the leading cause of death, was at least 50% higher among African Americans than Whites [1]. African Americans, Latinos, and those who are poor, less educated or disabled experienced a higher prevalence of diabetes and obesity, compared to their counterparts [1]. Risk of uncontrolled hypertension was higher among Mexican Americans, immigrants and the uninsured [2], and rates of HIV diagnosis were eight-fold higher among African Americans and two-fold higher among Latinos and Native Hawaiians/other Pacific Islanders compared to Whites [3]. Regional variations were also notable, with Southeastern African American and Whites experiencing shorter life expectancy than their counterparts from other U.S. regions [1]. Between 1992 and 2006, mortality rates among women increased in 42.8% of U.S. counties while only 3.4% of counties experienced mortality increases among men [4]. Sexual and gender minority (SGM) groups experienced higher incidence and mortality rates due to chronic disease, more depression, anxiety and suicidal ideation, and greater substance use than non-SGM groups [5]. Adults with a disability were more likely to have cardiovascular disease, be obese, be a current smoker, be physically inactive, and less likely to receive preventive health screenings than those without a disability [6].

To address these inequities, the National Institutes of Health (NIH) Health Disparities Strategic Plan recommends translating scientific discoveries including effective behavioral interventions to reach disparity populations, defined as subgroups that have worse health compared to the general population [7]. Evidence-based interventions (EBIs) are research-tested behavioral interventions found to be effective in achieving desired outcomes [8]. Most EBIs were developed and tested in academic settings for mainstream, highly selected populations. Fewer EBIs were designed for or have been applied in disparity populations, thus they are not reaching those most likely to benefit [9, 10].

To address health disparities, we need translational models/frameworks that can address the special issues of communities experiencing such disparities, and provide methodological guidelines to researchers. The NIH Stage Model describes six general stages of translation (from basic research to implementation), but does not provide methodological details for implementing interventions [11]. Another model, the Consolidated Framework for Implementation Research focuses on a taxonomy used to classify characteristics of the intervention, the inner and outer settings, individuals, and implementation processes, rather than specifying practical methodological steps [12].

We identified five models for disseminating EBIs into real-world settings from the fields of implementation science and public health that describe methodological steps. These include Intervention Mapping [13], the Center for Disease Control and Prevention’s (CDC) Replicating Effective Programs (REP) [14], CDC’s Division of HIV/AIDS Prevention (DHAP) Framework [15], the Exploration, Preparation, Implementation, Sustainment Framework (EPIS) [16], and the Evidence Driven Community Health Improvement Process (EDCHIP) [17]. Strengths of these frameworks are that most address multilevel influences on program implementation, emphasize late-stage translation in real-world settings, and attempt to balance scientific evidence with fit to the context. Some emphasize involving communities in the process [15–17]. However, none of these translational models focus on the special methodological issues pertaining to reducing health disparities or recognize the product as a new intervention. Methods for transferring scientific knowledge into programs to reduce health disparities have just begun to receive attention. Chinman and colleagues consider addressing disparities as a “special case” of implementation science and suggest blending methods from health disparities research and implementation science [9]. To reduce disparities in hypertension, Mueller and colleagues recommend “designing and testing pragmatic interventions in real-world settings using implementation research methods” (p. 712) [18]. Lopez-Class and colleagues argue that “late stage” translation research (T4), i.e., efficacy and effectiveness trials in community settings, holds potential for reducing health disparities [19]. These recent papers provide a starting point for systematic efforts to advance translation of EBIs to reduce health disparities.

### Why we need a new implementation science framework to address health disparities

Addressing health disparities requires delivering effective interventions that are acceptable, practical, and designed to address the health needs of at-risk populations. Translational models need to account for large differences between the original EBI conditions (academic setting, mainstream population, professionals as interventionists) and the health disparity population and community [9, 10].

Interventions need to be delivered in community as well as health care settings to reach disparity populations that tend to have limited access to health care. Translation models should account for community strengths and incorporate the resourceful solutions they have developed. The communities’ knowledge of the populations and the drivers of disparities can inform development of effective programs. To address resource constraints, interventions need to strategically build on existing infrastructure and develop new infrastructure to facilitate sustainability [20]. Efforts to bolster community resources and capacity increase the likelihood of success and sustainability [17]. To take full advantage of these resources and address disparities in a sustainable way, translational models need to incorporate methods for engaging communities throughout the entire process.

Regarding populations, one needs to consider the characteristics of the disparity populations and how they might differ from mainstream populations in which most EBIs have been tested. Key differences can include culture, language, English proficiency, literacy, poverty, health beliefs, access to resources, and others. Interventions resulting from this process need to be sensitive to these characteristics.

To address these issues, we need a paradigm that enables us to design and evaluate interventions delivered in real-world community settings *from the outset*. There are numerous EBIs that have been shown to be efficacious under optimal conditions. We can expedite the successful translation of these interventions to new settings and populations experiencing health disparities, but this process involves unique methodological steps. These steps help to ensure that the intervention reflects relevant scientific evidence (including EBIs) and simultaneously fits the target audience and community from the start.

In this paper, we propose a new paradigm that we refer to as “transcreation” for designing and implementing behavioral interventions specifically for communities experiencing health disparities. We present the Transcreation Framework for Community-engaged Behavioral Interventions to Reduce Health Disparities (referred to henceforth as the transcreation framework) that describes methods for designing, delivering, and evaluating interventions to reach disparity populations in community settings. Our methods are intended to address limitations of traditional research-to-practice models for translation of scientific knowledge, specifically when implementing behavioral interventions to reduce health disparities.

## Main text

### The transcreation framework

The term transcreation has been applied widely in the field of marketing. Among global marketing professionals, transcreation is recognized as the process whereby messaging and content are developed or adapted for an audience so that they resonate in local markets and yet deliver the same impact as the original [21]. Use of the term “transcreation” in the health arena has had a narrower scope, referring to the adaptation of health education materials for improved understanding and cultural relevance to specific language and ethnic groups [22]. We define transcreation as the processes of planning and delivering interventions to reduce health disparities so that they resonate with the targeted community, while achieving intended health outcomes.

The transcreation framework aims to guide researchers and community partners to reduce health disparities by developing and testing behavioral interventions that are grounded in scientific evidence and build on community strengths. It shifts the emphasis from adapting and translating one EBI for delivery in a new setting, to the design of a new intervention that better fits local needs and contexts. Our transcreation framework calls for research in which evidence building starts with interventions that are tested initially in community settings and disparity populations, rather than beginning with an efficacy trial conducted under optimal but constrained circumstances. Onken and colleagues suggest that this type of research lies somewhere between traditional efficacy research (tested using optimal conditions) and effectiveness research (tested in real world settings) [11]. Chinman and colleagues refer to this as a “hybrid” design that can accelerate the pace for translating EBIs into the real world [9].

**Fig. 1:**
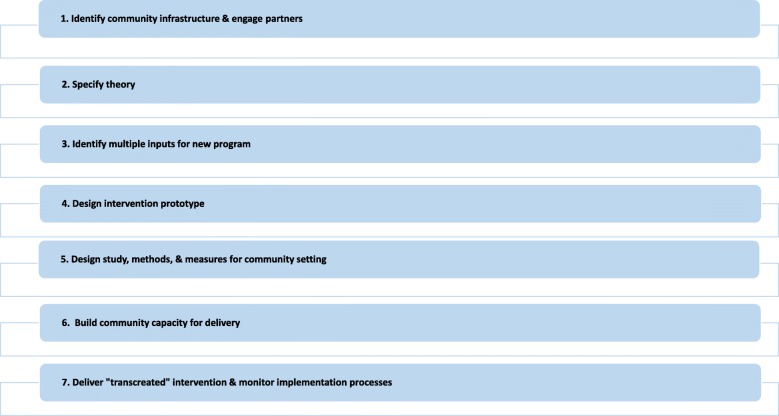
The Transcreation Framework: A Seven-Step Process. This figure depicts the seven steps involved in designing, delivering and evaluating behavioral interventions in communities to reduce health disparities

Our framework emphasizes scientific rigor, starting with theory, integrating scientific evidence in intervention design, utilizing scientific methods to evaluate effectiveness of interventions delivered in community settings, and applying stringent methods of evaluating implementation processes. It involves engaging communities throughout the research process - researchers work with community stakeholders to transcreate EBIs to be meaningful and deliverable with fidelity in community settings. This includes methods for capitalizing on community resources and building capacity by engaging community members in intervention delivery, and even in recruitment and evaluation. This strategy increases the likelihood that interventions will be culturally sensitive, appealing, and sustainable. The seven steps of the transcreation framework are listed in Fig. 1.

This framework builds on our prior methodological framework and training resource for adapting behavioral interventions to address health disparities [23]. We have made several major advances in our framework. We now include two types of theory: a socio-ecological theory of the determinants of the health disparity being targeted, and an appropriate behavior change theory. We place a greater emphasis on balancing fit to the context with fidelity to scientific evidence. We have incorporated methods for hiring and training community members as interventionists and study staff, including developing standardized intervention manuals. Finally, we specify methods for three types of evaluation: *formative* to develop the intervention, *process* to understand factors that affect implementation, and *summative* after the trial to inform future implementation and dissemination efforts. Perhaps the most important advance is shifting away from the concept of translation and adaptation of EBIs to transcreation, since it has become apparent to us that application of our framework results in a *new intervention designed specifically with community partners to reduce a targeted disparity.*

The revised framework incorporates our experience and research with several behavioral interventions to reduce disparities since that publication, as well as emerging literature on the intersection of implementation science and health disparities research [9]. Our own experience includes conducting community-based trials in multiple diverse minority and underserved communities experiencing health disparities (African Americans, Latinos, poor Whites, rural and urban, low-income cancer patients, and low-income individuals at risk for diabetes). We have tested several types of behavioral interventions (cognitive stress management, physical activity, and diet), using a variety of delivery modes (trained peers, groups led by public health practitioners, mobile phones, health coaches) that have improved health behaviors, physical and emotional well-being, and other outcomes [24–26].

Use of the framework assumes that an academic/community partnership has been established. Prior to using the framework, there should be a shared understanding of the specific health disparity that will be addressed by the partnership and intervention, e.g., higher prevalence of a risk factor in a disparity population group or lack of access to culturally competent services for a specific condition that are a shared concern. In Table 1, we present a detailed description of the seven steps. Although these steps are described sequentially, in practice, they may be iterative or occur at multiple time points in the transcreation process.

**Table 1:** Transcreation framework for community-engaged behavioral interventions to reduce health disparities

Step	Description and Methods
1. Identify community infrastructure & engage partners	
A. Identify optimal community settings	Identify settings with infrastructure for delivering and sustaining intervention, experience providing similar services, and that are convenient for the target population.
B. Identify partners and form an academic-community coalition to address the health disparity	Identify persons in the community with intimate knowledge of disparities and contributing factors and academic partners with relevant interest and experience; involve them in all phases of planning and execution. Develop an academic-community coalition based on CBPR principles (e.g., trust, shared decision-making, value scientific and community knowledge equally) *that is responsible for all subsequent steps*.
2. Specify theory	
A. Identify framework of social-ecological determinants of disparities	Identify, review, and select theoretical framework of determinants of disparities that fits the health priority and context. This involves using CBPR engagement principles.
B. Determine theoretical basis for behavioral changes	Working with the coalition identified in Step 1, identify, review and select one or more theories of behavior change relevant to the targeted health disparity population.
3. Identify multiple inputs for new program	
A. Identify scientific evidence relevant to planned intervention to address targeted health disparity	Review evidence-based interventions (EBIs), systematic reviews, evidence-based guidelines, and other evidence (e.g., optimal delivery modes) that can inform the intervention. For EBIs, identify core components that reflect mechanisms of action.
B. Obtain input from community on locally developed programs, community resources, and target population (formative evaluation)	Review locally developed programs relevant to targeted disparity.
	Identify cultural, practical, organizational, and contextual factors affecting intervention design, success, and sustainability through formative research including community resources, population characteristics and needs, and knowledge of determinants of disparity.
4. Design intervention prototype	
A. Design intervention to incorporate scientific evidence and locally developed programs.	Examine commonalities of EBIs and other scientific evidence and locally developed programs and synthesize.
	Build in intervention components and delivery methods that are supported by scientific evidence; include components that address hypothesized mechanisms of action.
B. Design intervention for fit to community setting and population	Build consensus on fit of potential intervention to community and potential to address targeted disparity. Assure that intervention is practical, accessible, and can be delivered within existing resources or with some capacity building.
	Incorporate content to fit population including culture, language, and learning styles, and format to fit reading level and preferred communication channels.
C. Integrate 4A and B to develop intervention components; vet prototype for relevance and potential for success.	Design specific components and content by weighing tradeoffs between scientific evidence and fit to setting (community, population).
	Specify delivery format (e.g., phone, in-person, group), location of in-person sessions, who delivers the program, session format/content, and dose.
	Document components and rationale for each; have key stakeholders review prototype, modify as indicated.
D. Manualize intervention to assure standardization	Develop detailed program manual for interventionists that specifies program delivery components, content, and procedures. Manual provides guidance to interventionists on methods to increase the fidelity of program delivery. Develop participant manual using principles for low-literacy participants.
5. Design study, methods, & measures for community setting	
A. Develop rigorous study design that is appropriate for intervention delivered in community setting	Randomized controlled trials, especially individual-level, may not be the most appropriate design. Identify alternatives that retain scientific rigor, e.g., cluster randomized trials, pragmatic trials, rigorous quasi-experimental designs.
B. Develop outreach, recruitment, and data collection strategies appropriate for population and setting	Design strategies based on evidence of effectiveness in disparity populations and perspective of community.
	Utilize community members for outreach, recruitment, and data collection in community settings to the extent possible; develop training protocol.
C. Select measures of outcomes, mediators, and moderators that are relevant and appropriate for population	Select outcomes based on conceptual framework linking components to outcomes. Identify measures that are responsive to similar interventions. Identify measures of moderators and mediators of effects. Assure that all measures are appropriate for the target population and meet stringent psychometric criteria in that population.
6. Build community capacity for delivery	
A. Enhance infrastructure and expertise	Compensate community organizations and members for research involvement. Train community organization staff on skills that can be applied to fund, deliver, and evaluate programs in the future.
B. Select/train community-based interventionists (an interim intervention)	Establish qualifications for community-based interventionists (e.g., CHWs) including desired level of competence/knowledge after training. Hire and train community-based interventionists. Utilizing interventionist manual developed in step 4D, create training protocol including: 1) content, format, theory, and protocol of “transcreated” intervention, 2) delivery skills including communication skills, handling problems, and 3) importance of fidelity to the protocol.
7. Deliver “transcreated” intervention & monitor implementation processes	
A. Create and implement methods for monitoring delivery of intervention and providing ongoing technical assistance to interventionists	Create ongoing technical assistance plans for interventionists as they deliver the intervention. Develop structured assessment for monitoring fidelity of interventionists to protocol. Establish system for providing feedback and support to interventionists if needed to improve adherence or prevent burnout. Establish system for modifying intervention if needed to address unanticipated situations.
B. Create and implement methods for assessing other processes of delivering intervention (summative evaluation)	Design specific procedures and data collection strategies to assess implementation processes. Interventionists: Suggested improvements, difficulties delivering program, acceptability of training and manual. Participants: Real-time - Program receipt (attendance, how well they learned components) and enactment (can demonstrate skills). Retrospective - perceived benefits of program, suggested improvements, perceived usefulness and ease of use of program components and materials. Stakeholders such as program managers, executive directors: issues in implementing program in that setting; successes and challenges of implementing program, suggestions for improvement.

### Step 1: Identify community infrastructure and engage partners

Transcreation research builds on existing community infrastructure and expands it for implementation. Step 1A is to identify intervention delivery settings and academic partners that share a targeted health priority and have appropriate infrastructure and experience. The composition of the academic-community coalition that is formed in this step will vary depending on the health priority being addressed, community needs, infrastructure and the ability to convene groups and individuals [17]. This infrastructure might include organizations or public health departments focused on chronic diseases and academic institutions. Settings serving disparity populations can include community-based organizations, government entities, community-based health care clinics, senior centers, public health departments, faith-based organizations, and other systems. Optimal settings can include those already delivering an intervention to address the targeted problem, e.g., to develop a stress management intervention for Latinas with breast cancer, we partnered with a community organization that provided cancer support programs for low-income Latinos [27].

Step 1B focuses on identifying individuals who are stakeholders with deep knowledge of the targeted health disparity and population and who are interested in implementing an intervention to reduce the targeted disparity. Partners can include multisector representatives, e.g., administrators, staff members, clients, community residents, and civic and private sector partners.

Partners form an academic-community coalition that is based on community-based participatory research (CBPR) principles (e.g., trust, shared decision-making, equal value placed on scientific and community knowledge) *and is responsible for all subsequent steps*. Using CBPR principles of engagement, partners will be engaged in all phases of research including providing inputs to program design, formative research, creation and vetting of a prototype, implementation processes, summative (post-implementation) evaluation, and dissemination of results and successful programs [28]. Partners need to be compensated through direct funding in proportion to the time and effort expended on the project. Employing CBPR principles to ensure equitable partnerships throughout and remunerating community partners and stakeholders helps build trust over time and ensures that ongoing community input is integrated and culturally appropriate approaches are discussed [28].

### Step 2: Specify theory

Beginning with a strong theoretical basis for the targeted disparity and intervention aids in understanding mechanisms of action [29], assuring that core components are included. Step 2A focuses on theoretical frameworks specifying social-ecological determinants of health, health behavior, and health disparities [19, 30, 31]. Academic researchers and community stakeholders work together to identify, review and select appropriate health disparities and health behavior change theories and frameworks [28]. Selection of these frameworks will be based on their relevance to the health priority, population and context. For example, working with community partners in one of our studies, we identified important determinants of poor emotional well-being in the target population as marital/family discord due to miscommunication and financial hardship, so these became part of our larger health disparity framework. Selection of the appropriate theories is based on both scientific and community data and knowledge about the health priority and population. Western-based theoretical approaches can be blended with indigenous-based theories [28].

Step 2B is to apply theories of behavior change that account for individual and social factors; this assures that the intervention will be theory-based. For example, Social Cognitive Theory [32] has been applied in behavioral interventions for disparity populations [33, 34], because of the potency of its principles of self- monitoring, goal setting, and problem solving that can increase self-efficacy, which often mediates improved outcomes. Delineating the theory underlying the program may include a logic model describing how the program works. A compendium of health behavior change theories can guide these discussions [35].

### Step 3: Identify multiple inputs for new program

Two basic types of inputs to the planning process include scientific evidence and input from the community including locally developed programs and knowledge. Step 3A includes identifying and reviewing scientific evidence, which can include EBIs as well as other types of evidence. EBIs can be identified via articles and reviews, meta analyses, and websites. Of importance is to determine whether any minority or other disparity groups were included in EBI testing. It is helpful to secure program materials for candidate EBIs via websites or by contacting program developers. For each EBI, identifying core components that reflect specific mechanisms of action is key [11]. This should result in a conceptual framework of how each component leads to hypothesized outcomes, i.e., pathways/key mechanisms of effects.

Other types of scientific evidence can be as important as EBIs, including systematic reviews and evidence-based guidelines. Examples include evidence-based recommendations about community preventive services and interventions to improve health [17], and a synthesis of evidence on the role of self-efficacy for achieving optimal outcomes of self-management interventions [36].

Of special importance to health disparities is the sizable amount of scientific evidence supporting the effectiveness of interventions delivered by community health workers (CHWs). CHW interventions can improve preventive behaviors and health outcomes in disparity populations, most notably in under-resourced areas. Systematic reviews of CHW interventions have substantiated their effectiveness in improving glucose and lipid levels, systolic and diastolic blood pressures, physical activity, dietary behaviors, and mental health outcomes [37, 38]. CHWs can enhance intervention effectiveness and reach because they are trusted by and share the experiences of individuals in disparity communities. Evidence on the value of the roles of other community practitioners (e.g., social workers, community health care providers) needs to be considered.

Step 3B is to use formative research to obtain input from the community and the disparity population to be served to understand cultural, practical and contextual factors that might affect intervention design, implementation, and sustainability [39]. Community stakeholder inputs include locally developed programs, deep knowledge of the specific health disparity, frameworks and measures, and organizational and community resources that can be harnessed to increase the likelihood of success and sustainability [28]. Interventions that build on existing programs and resources will enhance the fit of the intervention to the setting. A review by the academic-community coalition of organizational structures, staff, skills, and inter-organizational networks within which agencies deliver interventions helps identify resources (e.g., community asset mapping). A thorough assessment of the target population, its risk profile, and determinants of health disparities that incorporates the knowledge and experience of local community members and practitioners is needed. Formative research can include focus groups and semi-structured interviews with community key informants.

### Step 4: Design intervention prototype

Intervention design involves the academic-community coalition synthesizing/integrating scientific evidence and community input (inputs identified in Step 3) into a program description. Step 4A is to assure that the intervention reflects relevant scientific evidence. By including core components of EBIs, program content can address mechanisms while integrating other scientific evidence. For example, a peer-delivered stress management program for Latinas with breast cancer incorporated components reflecting known mechanisms for improving quality of life, namely, strategies to enhance self-efficacy for managing cancer, and peer support from a Latina cancer survivor [27]. Use of peer delivery was based on evidence of the effectiveness of CHWs in diverse communities and on a specific peer support model developed by one of the partner community agencies. In another study, evidence that action plans were effective in improving self-efficacy [40] was the basis for using them in a diabetes risk reduction intervention for lower-socioeconomic status and minority adults [33]. When translating interventions for sexual and gender minority older adults, Fredriksen-Goldsen and colleagues suggest including components directly addressing the higher rates of relevant risk factors in these populations (e.g., stigma, discrimination, victimization), which also need to be included in the relevant health disparity framework [41].

Step 4B includes designing the intervention to fit the population and community, and involves utilizing program delivery methods, staffing models, and community assets identified in Step 3B. Again, this involves iterative processes by the academic-community coalition members to review all candidate sources of knowledge and scientific evidence thus far, until consensus is reached on core components. Materials and strategies need to accommodate cultural and language/literacy issues, convenience, and accessibility, to improve participation. For example, knowing that the target population has limited transportation and discretionary time can result in a design in which group-based components are delivered in neighborhood settings to increase access [33]. Step 4B can involve applying methodological frameworks for culturally adapting interventions such as identifying messages that support ethnic practices or the unique contexts of persons with disabilities [42]. Low-literacy materials and visual aids can improve knowledge among individuals with a range of literacy levels [43]. Regarding fit to the community, practical issues can affect implementation, adoption and sustainability [44], requiring strategies to address resource needs. For example, to reduce costs, translation of a caregiver support program for Area Agencies on Aging resulted in fewer sessions than the original [45].

Step 4C is for the coalition to integrate 4A-B into a prototype and vet the components. The integration can involve comparing form (key elements/components) and function (mechanisms and processes that lead to desired outcomes) of the EBIs [46]. Such an approach allows “context level adaptation” of the intervention form, e.g., varying the literacy level of materials, while preserving the function, e.g., mechanisms of action specified by the intervention’s underlying behavior change theory [46]. The academic-community coalition considers and decides together what aspects of, and how, EBIs need to be adapted to maximize potential effectiveness and adoption, while preserving their scientific integrity. Typically, for interventions aimed at reducing health disparities, this step is heavily influenced by community members and stakeholders that include individuals struggling with the specific disparity being targeted.

Nápoles and colleagues describe the entire process of integrating an EBI tested in white women, a locally developed program for Latinas, and formative research to create Nuevo Amanecer, a stress management intervention for Latinas with breast cancer [27]. Mejia and colleagues describe a variety of programs that have resulted from processes of adapting EBIs to local contexts, ranging from minor surface structure changes to substantial adaptations [47]. In another case, they describe adapting evidence-based principles (functions) selected by community stakeholders from a number of EBIs, rather than the standard model of adapting a single EBI [47]. This step may result in a new program that “may bear little resemblance to the original EBI as local consumers make decisions as to what aspects of existing EBIs they deem relevant to their local communities” (p. 691) [48].

Step 4D is to operationalize the program by defining the specific components. Examples of components include small-group introductory session, written program manuals, one-on-one planning sessions, telephone counseling/coaching, audio-visual materials, and group workshops [27, 33]. Each component or session can be described in terms of its objectives, content, format (e.g., phone, in-person), timing, dose (number and duration of sessions), location, and who delivers it. The program description should include behavioral change strategies being used such as education, skills training, and motivational strategies [33, 34]. This step includes manualizing the intervention to assure standardization of key core components and functions. Standardization of the manual and materials provides a basis for training interventionists and assuring fidelity of delivery to the transcreated intervention [11]. Consultations with the community allow for vetting of program components and final adaptations.

### Step 5: Design study, methods, and measures for community setting

Step 5 focuses on specialized methodological strategies for evaluating the transcreated intervention, i.e., conducting experimental studies in community settings. Given that the transcreated program and the setting are new, it is critical to implement a stringent scientific evaluation of the intervention being delivered in a community setting where optimal control of study conditions is not possible. Traditional research methods usually require augmentation to be appropriate for ethnic minority and lower-socioeconomic status populations [49].

Step 5A concerns finding the most rigorous study design that is acceptable to the community and practical. Identifying acceptable control groups requires special consideration. Designs in which individuals are the unit of randomization may not be appropriate for community-based interventions due to practical concerns (e.g., health system-level changes that may not be under investigator control), ethical reasons (e.g., minority communities experiencing unequal treatment may resist the intentional withholding of a treatment to individuals), or scientific considerations (e.g., contamination). Implementation science uses a range of study designs that allow for causal inferences and approximate experimental designs, with tradeoffs between internal and external validity, e.g., group randomized trials, interrupted time series designs. For example, group randomized trials address potential bias by matching groups on stable correlates of the outcome, such as age, in contexts where individual randomization is culturally unacceptable or not feasible. An interrupted time series design is indicated when the specific time point that an intervention occurs is known, longitudinal outcome data are available for a period of time before and after intervention onset, and randomization is not feasible. Interrupted time series allows assessment of trends before, during and after implementation [50]. Excellent overviews of the issues involved and alternative designs are available [50, 51].

Step 5B pertains to development of study methods such as recruitment and data collection that provide scientific rigor and also meet community needs. Because convenience for participants implies conducting recruitment and data collection in the community, methods need to be well designed and clear, including monitoring to assure consistency with protocols. There is much literature on recruitment of disparity populations. Strategies with demonstrated effectiveness include using a conceptual framework of determinants of recruitment to guide efforts and engaging community change agents [52]. For studies targeting participants with specific risk factors, one strategy is to conduct community-based health education and screening and then recruit those identified as “at risk” [53]. Step 5B includes identifying strategies for overcoming common barriers to data collection in the community. For example, we developed language and literacy-level appropriate videos demonstrating self-collection procedures for repeated salivary samples to assess diurnal cortisol rhythms among rural Latinas with breast cancer. Conducting data collection in the community is feasible and increases retention of disparity populations, but requires considerations such as obtaining space in community centers and having portable data collection materials or home-based assessment technologies [25].

Step 5C focuses on identifying outcome measures that are responsive to change, linked specifically to intervention components, and appropriate for the population. Once academic and community partners have worked together to identify the health priority and population being targeted, the health disparity and health behavior frameworks that will guide measures selection, and the appropriate intervention components that are culturally appropriate, feasible and acceptable, outcomes are selected, or developed and pretested if no relevant measures exist. This occurs through a review of candidate measures, discussion of alternatives, and co-selection of final measures so that they will be sensitive to changes targeted by the intervention and can be collected in community settings given resource and time constraints.

Selecting measures requires attention to hypothesized mechanisms, thus a mechanism and its measure should be stated for how each component is linked to a desired outcome. A peer-delivered stress management program for Latinas with breast cancer included components to increase social support and self-efficacy, thus measures of social support and self-efficacy were used to evaluate these as mediators of change [34]. The theoretical framework underlying an intervention may also point to the need to measure potential moderators of intervention effects, e.g., characteristics of subgroups that may respond differentially to treatment effects.

All measures need to be culturally appropriate and meet stringent psychometric criteria in the targeted populations [54]. This involves defining concepts from the perspective of the target population, locating measures of those concepts, reviewing whether measures reflect the concept and have good psychometric properties in the target population, and choosing the best measure(s) and adapting them if needed [54]. Measures may need to be culturally adapted, translated into other languages, and pretested using cognitive interviewing methods. Measures may need to be modified or simplified in format to accommodate limited literacy or low socioeconomic status. It is important to obtain written permission to use or modify existing measures.

### Step 6: Build community capacity for delivery

Building capacity focuses on enhancing community infrastructure (identified in Step 3B) by developing a community-based workforce that can blend community knowledge with scientific principles. Community staff can obtain training in research and evaluation methods, while researchers gain knowledge of a community’s best practices, assets, and processes of implementing successful programs. Together, this shared exchange of information enhances opportunities to expand transcreation of effective programs.

Step 6B is to identify and train qualified individuals from the community (practitioners, CHWs, community members who have experienced the health problem being addressed) who can deliver the intervention. Hiring interventionists from the community promotes cultural sensitivity and trust, and builds community capacity [55]. Training these interventionists is an intervention in itself, requiring a training protocol and evaluation, as well as ongoing technical assistance (step 7A) to assure competency. Training community interventionists involves using active learning strategies (e.g., role playing and modeling) and detailed interventionist and participant program manuals [45]. Training may be needed on organizational and delivery skills, communication skills, role playing, role modeling desired behavior change, troubleshooting implementation problems, maintaining confidentiality, and tracking program activities [56]. Training needs to cover special considerations when working with disparity populations, such as limited English proficiency, low literacy, and financial hardship. It is important to provide interventionists with information on community resources so they can make appropriate referrals. Interventionists need to learn how to track participant receipt of the intervention (see step 7), e.g., attendance, receipt of components, and problems experienced by participants with program activities. To assure adequate preparation to deliver the intervention, core competencies need to be defined and assessed [45]. Reducing health disparities usually requires training community practitioners and researchers in patient-centered research and CBPR approaches. For CHWs located in rural areas, videoconferencing can be used for training and technical assistance support.

### Step 7: Deliver “transcreated” intervention and monitor implementation processes

Step 7 involves having trained interventionists deliver the transcreated program to participants, and monitoring implementation processes. When testing the intervention first in a community setting, the process of monitoring its delivery, termed implementation fidelity [57], is crucial to its success due to the influence of factors outside of researchers’ control. We have built on the methods for monitoring these processes developed by the NIH Behavior Change Consortium [58], noting how these methods apply to health disparity interventions.

Step 7A is to design and implement methods for monitoring intervention delivery with feedback loops. Demonstrating that an intervention was delivered as intended (treatment fidelity) is important in any effectiveness trial, but is essential when interventions are delivered by trained community members, practitioners or clinicians. Structured fidelity assessments can occur through ratings of directly observed, audiotaped or videotaped sessions. This step includes plans for ongoing technical assistance and/or training to interventionists to assure fidelity and prevent unanticipated implementation challenges [57]. Interventionists need a way to contact researchers or community partners to report problems and obtain help or suggestions. Regular phone calls with interventionists can help. An evaluation of a church-based nutrition program for African Americans noted the importance of providing ongoing technical assistance to assure effectiveness [59]. Issues can be substantive (procedures for dealing with extremely depressed participant) or logistical (difficulty reaching participants). Researchers and community partners can provide help in the form of ideas or resources that are available. For example, similar to our experiences, community interventionists reported needing more information on how to manage participants who were not attending sessions [56]. In some situations, problems may necessitate modifying the intervention protocol.

Should fidelity assessments identify concerns, this step includes a plan to provide sensitive yet direct feedback to interventionists to improve protocol adherence. A feedback mechanism can improve practitioners’ performance and inform ongoing coaching and supervision [57]. A foundation of trust between academic and community partners is needed when academic personnel provide feedback to interventionists who are not directly employed by the study. Community organizations often retain control of transcreated programs, thus need to feel engaged and responsible for insuring that the program is implemented according to protocol.

Step 7B is to create and implement methods for monitoring an array of intervention delivery processes. These include quantitative (structured surveys, administrative tracking data) and qualitative (direct observation, open-ended interviews) mixed methods approaches in real time or retrospectively. Sources of information include interventionists, participants, stakeholders, and observers. Community partners and stakeholders are involved directly, providing feedback and troubleshooting alternatives with academic partners*.* Results can be triangulated across methods and sources. Some of these measures of intervention implementation need to be study specific to capture relevant indicators of implementation, e.g., participant mastery of key content. General approaches include monitoring dropouts and their reasons. Semi-structured interviews of participants can assess the perceived usefulness and ease of use of specific components, and suggested improvements, while probing for in-depth understanding of complex contextual influences on program implementation [24]. Mixed methods can be used to assess interventionists’ perspectives on practical and cultural factors affecting participation, acceptability of training and manuals, and perceived program effectiveness, For example, interventionists delivering a lifestyle intervention to overweight older rural participants identified self-monitoring (e.g., starting each session with a weigh-in) as important to program success [56].

Stakeholders such as administrators, program directors, and staff from the community settings hosting the intervention can provide “system-level” feedback [44]. This can be done regularly throughout the implementation to enable course corrections, such as reducing the amount of telephone support provided to participants due to organizational burden.

## Conclusions

The Transcreation Framework for Community-engaged Behavioral Interventions to Reduce Health Disparities includes the major advantages of other implementation science frameworks while adding important methodological steps critical for reducing health disparities. Perhaps the most novel feature is recognizing that when researchers address social determinants and health disparities with the full engagement of community partners *experiencing the disparity,* the intervention produced is not an adapted EBI, but a new intervention because of the extensive adaptations required to fit the community context in the presence of these disparities.

We discuss five defining features of our framework: 1) balances fidelity to scientific evidence and fit to develop a new “transcreated” intervention, (2) tests the transcreated intervention in community settings, 3) engages the community throughout and builds capacity, 4) uses rigorous scientific methods in community settings, and 5) expands the types of evidence used for transcreation of behavioral interventions. We also highlight differences between our framework and the other implementation science models that we reviewed.

### Balances fidelity to scientific evidence and fit to community setting

Our framework includes attention to treatment fidelity as well as focusing on the fit of the intervention to the community and testing the intervention in the community from the outset. Our approach shifts the focus from translation of a single tested EBI to the synthesis process we call transcreation in which varying stakeholders build new programs that retain the core components of successful programs and community knowledge, while making adaptations that improve the fit to local contexts. A major difference between our framework and the other implementation frameworks with methodological steps that we reviewed is that they view the resulting program as a translation or adaptation of one EBI, and not a new program that integrates various EBIs and other types of evidence, along with community knowledge.

### Tests the transcreated intervention in community settings

Our framework calls for research in which interventions are tested initially in community settings and disparity populations rather than beginning with an efficacy trial conducted under optimal but constrained circumstances. This approach means focusing on later stages (T3 and T4) of translation to circumvent the extensive and iterative adaptations that might result as researchers proceed through earlier translation stages (T1 and T2). As stated by Onken and colleagues, “The intervention development process is incomplete until an intervention is optimally efficacious and implementable with fidelity by practitioners in the community” (p. 22) [11]. An important consideration in late stage translation is that community partners are the foundation and need to be funded for these efforts. Broad community involvement on the part of affected communities was emphasized in only two of the five frameworks that offered methodological steps [13, 17].

### Engages the community throughout and builds capacity

Primarily, our model differs from other translation models in that we propose starting the transcreation process with the full integration of the community’s knowledge, local programs, and participants from the beginning, which is necessary to address social determinants of health and maximize feasibility. This approach recognizes that EBIs may not demonstrate the same level of efficacy as originally tested unless we include community members and practitioners in ensuring their fit to local contexts. Our framework adheres to CBPR principles and integrates these with implementation processes. Other implementation frameworks tend to include community representatives in limited or early stages of intervention adaptation to design and pretest the program prototype. Similar to two of the models with methodological steps that we reviewed, we emphasize building on existing community resources such as locally developed programs and services, thus increasing sensitivity to the local culture and the likelihood of sustainability [15, 16]. This includes training qualified community members or practitioners as interventionists or as research staff (helping with recruitment and data collection). With ongoing training and technical assistance, this unique feature builds community capacity.

### Uses rigorous scientific methods in community settings

Our model stresses the use of rigorous scientific methods for conducting effectiveness trials, outreach and recruitment, and process evaluation in community settings. Although the methods used in effectiveness trials have been increasingly applied in health disparities research, they have not focused on the unique methodological considerations when these are applied in populations experiencing health disparities. The most prominent models either focus on engaging communities in the process of implementation and capacity building to address multilevel determinants of behavior, [14–17] or emphasize health behavior theory, core components, and mechanisms of action [13]; none address all of these features or health disparities frameworks. Also, we feature prominently state-of-the-art methods for monitoring implementation, sometimes referred to as process evaluation or summative evaluation. We have emphasized the need for carefully designed yet practical qualitative and quantitative strategies that fit the context. Formative evaluation serves to secure input from experienced stakeholders prior to and during transcreation processes, while summative evaluation assesses implementation processes to inform subsequent implementation efforts. Such evaluation is critical, especially when EBIs are being tested in vulnerable populations typically underrepresented in clinical research [9]. Three of the five implementation science models that specify methodological steps mention stakeholder input at different stages, but most often stakeholders tend to be agency planners or delivery system representatives, and not the community individuals faced with the health disparities; these frameworks also do not emphasize the greater need for rigorous evaluation in the context of health disparities settings because adaptations tend to be extensive [13, 14, 16].

### Expands the types of evidence used for transcreation of behavioral interventions

Our model redefines the term “evidence” in “evidence-based.” The term “evidence-based” usually is applied to describe research-tested programs for which there is empirical evidence that they improve outcomes. This narrow interpretation of “evidence” impedes our ability to reduce disparities because it discounts the equally valuable evidence and knowledge that public health and community sectors contribute. We encourage health disparities research that integrates evidence from systematic reviews, community guidelines, and best practices. Only two of the five implementation science frameworks that specify steps consider these additional sources of evidence [13, 15] and they assume that only one EBI is being translated or do not specify that components from more than one EBI can be utilized.

### Implications for reducing health disparities

Progress on reducing health disparities in the U.S. has been slow due to growing income inequality, systemic discrimination, and unequal access to resources, as well as ineffective translation models. Achieving population-level reductions in health disparities will involve concerted efforts that focus on *where we intervene, how we intervene*, and *how we sustain* effective programs. Based on emerging literature and our experience, we note some research and policy implications of our framework for reducing health disparities through transcreation research on behavioral interventions.

#### Accelerate progress toward reducing health disparities by using transcreation and beginning with later translation stages

Community-engaged transcreation takes behavioral interventions to the communities where they are needed. Applying our transcreation framework in the communities experiencing pernicious health disparities can enhance the design of interventions to maximize fit to the population, while preserving the scientific basis of behavioral interventions, and rigorously evaluating program outcomes and implementation processes. The goal is to create programs and design implementation strategies that reduce health disparities. Once programs and implementation strategies for delivery in community settings are shown to be effective, they can be scaled up and disseminated.

#### Build community capacity to address health disparities through transcreation of behavioral interventions

Embedding programs within existing community services and health care delivery networks can extend their reach and acceptability in communities with disparity populations. Implementation strategies that build on community assets and infrastructure maximize potential scalability and impact. Engaging communities that are experiencing disparities at each stage of the translational process “considers culture and diversity from a community-engaged research perspective” (p. 115) [20]. Training community practitioners or community health workers on cutting-edge EBIs and evaluation methods can enhance their ability to transcreate effective programs for local communities.

Enhanced training of CHWs to deliver effective interventions across a broad range of health issues is an excellent strategy for building capacity (see Step 3A) [34, 60]. Studies have supported the cost-effectiveness of CHW interventions [61]. CHWs are well-suited for providing higher intensity services needed to reduce disparities in highly impoverished populations [62]. They can be especially effective when integrated into health care systems with access to health care teams and electronic health records [62]. Due to widespread adoption of smartphones and internet access by minority populations, technology-enhanced CHW interventions may help address time, cost, and transportation barriers evident in disparity populations and allow interventions to be interactive and individually tailored for greater patient activation and effectiveness [63]. More research is needed to identify populations for whom approaches using mobile technology tend to be most effective, and the types of additional support needed to maximize their effectiveness in specific disparity populations.

#### Funding models that support sustainability of community programs

One of the biggest challenges to reducing health disparities by harnessing the potential of transcreated community-engaged interventions is sustainability funding. Sustainability funding and reimbursement opportunities are practically non-existent. State-level programs that utilize innovative funding models could significantly reduce disparities if implemented more broadly, but they require substantial resources and concerted effort to set up, and may require special legislation [64]. For example, the state of Montana partnered with Medicaid to reimburse community sites delivering an adapted Diabetes Prevention Program, achieving excellent enrollment and weight reduction targets [10]. The state of Delaware financed universal community-based screening, treatment, and patient navigation for colorectal cancer, resulting in the elimination of statewide disparities in colorectal cancer screening, incidence, and mortality between whites and African Americans [65]. In New Mexico, use of Medicaid-funded CHWs to provide navigation services to complex, high-need patients has been cost-effective, reducing emergency room visits, hospital admissions, and prescriptions [64]. Greater community- and population-level funding of effective transcreated programs could help reduce health disparities.

Our failure to identify effective implementation strategies of proven interventions contributes to the persistence of health inequities. Conversely, identifying highly effective implementation methods that consider the social determinants of health and community-based solutions may contribute significantly to achieving health equity. Working together, scientists, community practitioners, community organizations, and consumers can develop and test transcreated programs that are sensitive to contextual factors in communities experiencing disparities, build on and strengthen community assets, and are practicable and acceptable. Detailed evaluation of transcreated program processes and outcomes using rigorous methods can identify components and contextual factors that lead to desired behavioral changes and health improvements. Such methods for implementing behavioral interventions in real-world communities harness scientific evidence and community knowledge, apply it directly in community settings, and build community capacity to implement and evaluate interventions, thereby accelerating progress in reducing health disparities.

## Abbreviations

CBPR: Community-based participatory research
CDC: Centers for Disease Control and Prevention
CHW: Community health worker
EBI: Evidence-based intervention
HIV/AIDS: Human immunodeficiency virus/ acquired immunodeficiency syndrome
NIH: National Institutes of Health
T1 research: Translation to humans
T2 research: Translation to patients
T3 research: Translation to practice
T4 research: Translation to population health
U.S.: United States
